# Epidemiology and Retrospective Analysis in Extrapulmonary Neuroendocrine Carcinoma

**DOI:** 10.7759/cureus.12748

**Published:** 2021-01-17

**Authors:** Ashish Sethi, Mohammad Islam, Raj Moses, Gene Finley

**Affiliations:** 1 Hematology and Medical Oncology, Allegheny Health Network, Pittsburgh, USA

**Keywords:** poorly differentiated neuroendocrine carcinoma (pdnec), grade 3 neuroendocrine carcinoma, extrapulmonary neuroendocrine carcinoma (epnec), neuroendocrine carcinoma (nec)

## Abstract

Objectives: We conducted a retrospective analysis of patients with extrapulmonary neuroendocrine carcinomas (EPNECs) to explore the distribution and overall outcomes by different regimens and their primary sites.

Setting: We reviewed the outcomes of one of the largest data sets of patients with extrapulmonary small cell carcinomas (EPSCCs) identified at Allegheny General Hospital located in Pittsburgh, Pennsylvania, USA.

Participants: Patients diagnosed with grade 3 EPNECs were retrospectively identified.

Primary endpoint and epidemiology: Overall survival (OS) with different treatment regimens was the primary endpoint. Also, epidemiological factors such as risk factors, race, family history of cancer, and associated comorbidities were recorded.

Results: OS was 16 months in seven patients who received cisplatin/etoposide chemotherapy and 8.5 months in seven patients with carboplatin/etoposide chemotherapy. The commonest primary site was the gastrointestinal tract (GIT). Smoking history association was observed to be 50%. Merkel cell carcinoma (MCC) patients had significantly better OS. Simultaneously, an extensive form of disease pattern was also noticed in 94.4% of the patients. Significantly, neutropenic sepsis was observed in 71.4% of the patients who were treated with cisplatin/etoposide combination.

Conclusion: EPNECs demonstrated a low response rate to chemotherapy and high rates of distant metastases. Conclusively, brain metastases were rare.

## Introduction

Poorly differentiated neuroendocrine carcinomas (PDNECs) or high grade 3 type, also called small cell carcinomas (SCCs), is considered one of the most aggressive forms of malignancy arising from the epithelial tissues. Neuroendocrine carcinomas (NECs) can affect any organ system in the body, and most of these cases occur primarily in the lung. Extrapulmonary neuroendocrine carcinomas (EPNECs) refers to NECs occurring in any organ type except lung, *viz* gastrointestinal tract, genitourinary system, skin, bone, and pericardium. Only 5% of SCCs are extrapulmonary, with incidence rate ranging between 0.1% to 0.4% [1]. PDNECs account for as much as 10% to 20% of malignant digestive neuroendocrine neoplasms (NENs) [2]. Most EPNECs present with widespread metastatic disease at the time of diagnosis and have a poor prognosis with a median survival duration of 34 months in localized disease, 14 months in regional disease, and five months in distant disease [3].

Treatment such as chemotherapy with etoposide and platinum is considered the preferred (or standard) first-line treatment for both metastatic and localized NECs. Localized disease can also be treated surgically, if possible, or with chemo-radiation. It has also been noticed that the incidence of brain micrometastases in small cell lung cancer (SCLC) is high ~10% in comparison to EPNEC. Thus, it necessitates prophylactic cranial irradiation in the SCLC.

## Materials and methods

We conducted a retrospective analysis of clinical outcomes in 18 patients diagnosed with grade 3 EPNECs between January 1, 2018, and May 27, 2020, at Allegheny General Hospital located in Pittsburgh, Pennsylvania, USA. The study received approval from the Internal Review Board. Medical data were extracted from the Epic system. Overall survival (OS) with different treatment regimens was the primary endpoint, and epidemiological factors such as risk factors, race, family history of cancer, and associated comorbidities were recorded. Grade 3 or PDNECs were delineated based on histological features with >20 mitoses per 10 high power field (HPF) or Ki-67 labeling index >20%. Patients were divided into metastatic or non-metastatic disease based on clinical presentation.

## Results

Of the 18 patients, 50% were male. Our results demonstrated a median age of 63 years. The youngest individual was 29 years, while the eldest one was 85 years of age. Nearly 94.4% of NECs were observed specifically in the Caucasian race, and the majority of the presentation at the time of diagnosis was metastatic in nature, accounting for 94.4%.

A family history of cancer from our patient data was observed in 66.6% of cases. Another very likely causative factor was observed to be having a history of smoking, and the association was noticed in 50% of patients, whereas a history of alcohol consumption was noticeably present in 44.4% of the patients.

The most common location for EPNEC occurrence was observed in the gastrointestinal tract (GIT) in about 66.6% of patients. A noticeable involvement of the pancreas in GIT as a primary origin was maximum in 33.3% of patients. This was subsequently followed by the sigmoid colon in 25% of patients, and occurrence in the rectum was also recorded to be 25%. Stomach and common bile duct as primary origin sites were observed in one patient each. One case was diagnosed as metastasis of unknown primary origin. Genitourinary tract involvement as EPNEC was observed in 16.6% of patients. Ovary, as the primary site of origin, was involved in 66.6% of patients. We also observed two patients diagnosed with Merkel cell carcinoma (MCC), one with localized and one with extensive disease; both responded well to treatment and remain alive for 15 months and 25 months, respectively.

The liver was observed to be the commonest metastatic site (88.8%). One patient with extensive MCC was observed to have metastasis to bone and regional lymph nodes. Neutropenic sepsis was noticed in 71.4% of patients treated with the cisplatin-etoposide regimen in comparison to 28.5% with the carboplatin-etoposide combination group.

The most common regimen used in our study was the combination of etoposide-carboplatin and etoposide-cisplatin. OS was 16 months in seven patients who received cisplatin-etoposide chemotherapy and 8.5 months in seven patients with carboplatin-etoposide chemotherapy. Although the OS with cisplatin-etoposide was numerically better than carboplatin-etoposide, this was not statistically significant (p-value=0.29). Progression free survival (PFS) of 27 months with etoposide-carboplatin in one patient categorized as NEC of the ovary was seen. One patient in our study with NEC of the common bile duct received etoposide/carboplatin and atezolizumab combination based on IMpower133 trial with PFS of four months.

Pertinent findings regarding epidemiology, disease pattern, and treatment response are summarised in Tables 1-3.

**Table 1:** Demographic Features and Clinical Presentation GIT, Gastrointestinal Tract

Epidemiology Factors and Disease pattern	No. of Patients	Percentages (%)
Male Gender	9	50
Female Gender	9	50
Caucasian Race	17	94.4
Family History	12	66.6
Smoking History	9	50
Alcohol consumption	8	44.4
Metastatic or Extensive in nature	17	94.4
GIT Involvement	12	66.6
Pancreas	4	33.3
Sigmoid colon	3	25
Rectum	3	25
Genitourinary Tract Involvement	3	16.6
Ovary	2	66.6
Liver as Metastatic involvement	16	88.8
Neutropenic Sepsis with Cisplatin/Etoposide	5	71.4
Neutropenic Sepsis with Carboplatin/Etoposide	2	28.5

**Table 2:** OS and Epidemiology OS, Overall Survival: Length of time from either the date of diagnosis or the start of treatment for the disease.

Age	Race	Sex	Location of Primary Tumor	Distant Metastasis	Overall Survival (OS)
65	Caucasian	Male	Urinary bladder	Liver	13 months
56	African	Female	Pancreas	Liver	3.5 months
53	Caucasian	Male	Pancreas	Liver	5 months
53	Caucasian	Male	Rectum	Liver	24 months
59	Caucasian	Male	Pancreas	Liver	7 months
29	Caucasian	Female	Ovary	Liver	7 months
51	Caucasian	Female	Sigmoid colon	Liver	10 months
62	Caucasian	Male	Rectum	Liver	5 months
77	Caucasian	Female	Rectum	Liver	1.5 months
56	Caucasian	Male	Pancreas	Liver	4 months
70	Caucasian	Female	Sigmoid colon	Liver	14 months
85	Caucasian	Male	Sigmoid colon	Liver	4 months
78	African	Female	Unknown Primary	Liver	33 months
64	Caucasian	Male	Gastric	Liver	48 months

**Table 3:** PFS and Epidemiology PFS, Progression Free Survival: The length of time during and after the treatment of a disease that a patient lives with the disease, but it does not get worse. NA: Not Applicable

Age	Race	Sex	Location of Primary Tumor	Distant Metastasis	Progression Free Survival (PFS)
64	Caucasian	Female	Ovary	Liver	27 months
67	Caucasian	Female	Ampulla of Vater/Common bile duct (CBD)	Liver	4 months
66	Caucasian	Male	Merkel cell carcinoma	NA	15 months
54	Caucasian	Female	Merkel cell carcinoma	Bone, Distant lymph node	25 months

## Discussion

NECs have been associated with hereditary syndromes such as Von Hippel Lindau syndrome, neurofibromatosis type 1, multiple endocrine neoplasia type 1 and 2 (MEN 1/MEN 2), and tuberous sclerosis complex; however, none of our patients had a suggestive history. Nonetheless, the family history of different cancer types was observed in 66% of cases in our study.

**Figure 1:**
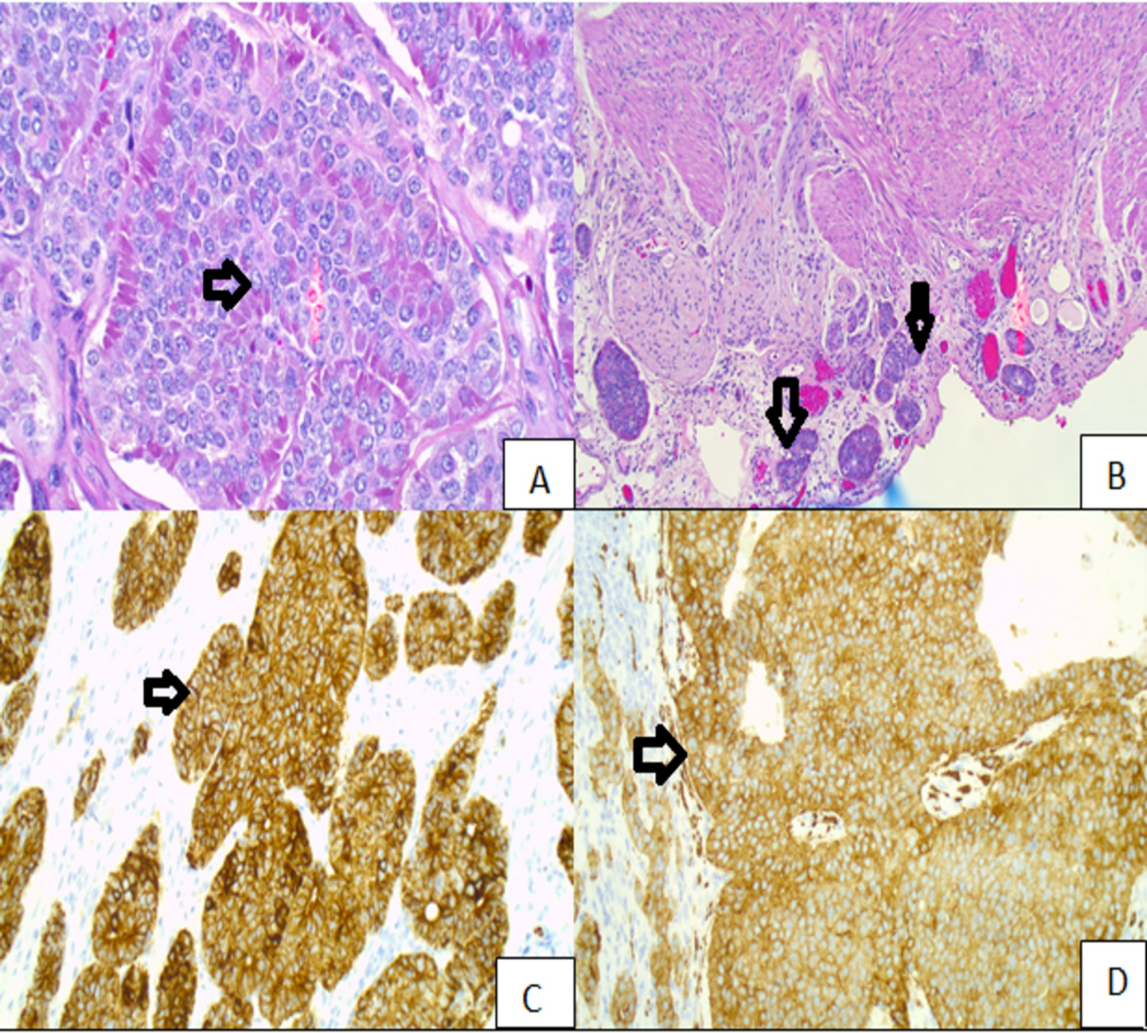
(A) NET. (B) NET invading visceral peritoneum. (C) Positive staining for pan-cytokeratin. (D) Positive staining for synaptophysin. NET, Neuroendocrine tumor. Permission was taken from the original publisher; adapted from Aslam et al. [4].

PDNECs and neuroendocrine tumors (NETs) are defined on the basis of expression of neuroendocrine markers such as chromogranin A and synaptophysin (Figure 1) with aggressive histologic features of PDNECs illustrating as high mitotic rate >10 mitotic figures/10 HPF, extensive necrosis, nuclear atypia, and the Ki-67 proliferation index, if performed, of more than 20% (Table 4)

**Table 4:** Neuroendocrine Morphology and Grade Classification NENs grade 3 represents two groups regarding the morphology and the grading: NECs and neuroendocrine tumor grade 3 (NET G3) [5].

Neuroendocrine Neoplasm	Morphology (Differentiation)	Grading G1-G3 (Ki-67 index in %)	Abbreviation
Neuroendocrine tumor Grade 1	Well-differentiated	G1 (≤2%)	NET G1
Neuroendocrine tumor Grade 2	Well-differentiated	G2 (3–20%)	NET G2
Neuroendocrine tumor Grade 3	Well-differentiated	G3 (>20%)	NET G3
Neuroendocrine carcinoma	Poorly-differentiated (large or small cell)	G3 (>20%)	NEC

Among 18 patients, 50% of the patients were men (nine patients), and out of them, approximately 60% of the affected patients had a history of smoking. Kim et al. have suggested no correlation between smoking and extrapulmonary small cell carcinomas (EPSCCs) [6]. 

In a similar study, focusing on primary NETs arising from the hepatobiliary and pancreatic regions, a response rate of 14% and median survival of 5.8 months were obtained in response to cisplatin plus etoposide [7]. In patients manifesting progressive disease after platinum-based chemotherapy, no standard alternate treatment is available [8]. Compared to the studies mentioned above, the average median OS in our patients was 8.5 months and 16 months for etoposide-carboplatin and etoposide-cisplatin, respectively. Further, in contrast to SCLC, topotecan as a second-line treatment option did not induce any remissions and resulted in a poor median PFS of only 2.1 months in a small subset of gastrointestinal NECs [9].

New advances in the management of small cell lung cancer with immunotherapy have shown great promise in recent clinical trials, such as the CASPIAN trial, which studied the effect of adding durvalumab to the standard therapy in patients with extensive stage small cell lung carcinoma (ES-SCLC) [10]. Durvalumab plus platinum-etoposide was associated with a significant improvement in overall survival, with a hazard ratio of 0·73 (95% CI, 0·59-0·91; p=0·0047) and median overall survival of 13·0 months (95% CI, 11·5-14·8) in the durvalumab plus platinum-etoposide group versus 10·3 months (95% CI, 9·3-11·2) in the platinum-etoposide group [10]. IMpower133 trial, which studied the use of azetolizumab in ES-SCLC, revealed longer OS in the atezolizumab group (median, 12.3 months; 95% CI, 10.8-15.9) than in the placebo group (median, 10.3 months; 95% CI, 9.3-11.3) [11].

Only one patient in our study with NEC of the common bile duct received IMpower133 and remains without progression four months into first-line therapy. None of the patients from our data list were enrolled for the CASPIAN trial as first-line therapy for EPNEC, and none of them received cranial irradiation during the treatment course.

Lastly, MCC, which also represents a high-grade EPNEC, is an aggressive form of skin carcinoma which can be localized or metastatic at the time of presentation. Surgical excision, chemo-radiation, and immunotherapy are considered as different management approaches for this subtype based on clinical presentation. However, a study done on advanced MCC resulted in an objective response rate (RR) of 56% with pembrolizumab as first-line treatment [12]. Prognosis of MCC is often poor and is dependent on the stage at presentation.

A number of biases may have influenced our results given the limited number of patients involved and lack of central pathology verification; we still observed certain pertinent inferences from our study. It was noticed that the association of smoking was relatively less compared to SCLC. Also, none of our patients had cerebral metastasis on magnetic resonance imaging (MRI). The pancreas as the primary site of origin was considered the most aggressive form of NEC, signifying poor prognosis with OS ranging between 3.5 months and seven months (Table 2).

## Conclusions

EPNECs is a rare entity with a variable response to platinum and etoposide. In our case series, the median survival was approximately one year. Cisplatin provided additional benefit at the cost of increased toxicity, mainly in the form of gastrointestinal toxicities and neutropenic sepsis. Also, many patients with co-morbidities or advanced age cannot tolerate cisplatin. The results of platinum-based chemotherapy are unsatisfactory, and clearly, new approaches are needed. Promising results in small cell lung cancer reported in the IMpower133 and CASPIAN trials may be a path forward. More research is needed to define the role of immunotherapy in EPNEC, expanding treatment options for both patients and physicians.
